# Correction: Exploring the potential antimalarial properties, safety profile, and phytochemical composition of *Mesua ferrea* Linn

**DOI:** 10.1371/journal.pone.0328758

**Published:** 2025-07-21

**Authors:** Atthaphon Konyanee, Prapaporn Chaniad, Arisara Phuwajaroanpong, Walaiporn Plirat, Parnpen Viriyavejakul, Abdi Wira Septama, Chuchard Punsawad

There are errors in the author affiliations. The correct affiliations are as follows:

Atthaphon Konyanee^1,2^, Prapaporn Chaniad^2,3^Arisara Phuwajaroanpong^2,4^, Walaiporn Plirat^2,3^, Parnpen Viriyavejakul^5^, Abdi Wira Septama^6^, Chuchard Punsawad^2,3^

1 College of Graduate Studies, Walailak University, Nakhon Si Thammarat, Thailand, 2 Research Center in Tropical Pathobiology, Walailak University, Nakhon Si Thammarat, Thailand, 3 School of Medicine, Walailak University, Nakhon Si Thammarat, Thailand, 4 School of Allied Health Sciences, Walailak University, Nakhon Si Thammarat, Thailand, 5 Department of Tropical Pathology, Faculty of Tropical Medicine, Mahidol University, Bangkok, Thailand, 6 Research Center for Pharmaceutical Ingredient and Traditional Medicine, National Research and Innovation Agency (BRIN), Cibinong Science Center, West Java, Indonesia.

The Funding statement for this article is incorrect. The correct Funding statement is as follows: This project is funded by the National Research Council of Thailand (NRCT) (Contract No. N41A670189) and the Walailak University Graduate Research Fund (Contract No. CGS-RF-2023/03). Additional support was provided by the Walailak University Ph.D. Scholarships for High Potential Candidates to Enroll in Doctoral Programs (Contract No. HP019/2021). The funders had no role in the study design, data collection and analysis, decision to publish, or preparation of the manuscript.
