# Supplementary material for: Exploring the potential antimalarial properties, safety profile, and phytochemical composition of Mesua ferrea Linn
Source: PLoS One. 2024 Dec 2;19(12):e0312047. doi: 10.1371/journal.pone.0312047 (PMC11611102; doi:10.1371/journal.pone.0312047)
Supplement: S1 File — (PDF) [file pone.0312047.s001.pdf]

Supporting information file 1.1

Data tables for graph generation

Table 1.1 Effects of the EMFL and EMFB on percentage parasitemia suppression in *P. berghei* ANKA-infected mice in a 4-day suppressive test. (Data presented corresponds to Figure 2)

Group	Dose (mg/kg)	% Parasitemia	% Suppression
Negative control group	-	49.80 ± 0.55[b,c,d,e,f,g,h,i]	0.00[b,c,d,e,f,g,h,i]
Positive control group (ARS)	6	2.18 ± 0.21[a,d,e,f,g,h,,i]	95.63 ± 0.42[a,d,e,f,g,h,i]
Positive control group (CQ)	25	1.00 ± 0.05[a,d,e,f,g,h,i]	97.99 ± 0.10[a,d,e,f,g,h,i]
Experimental group (EMFL)	200	29.31 ± 0.48[a,b,c,e,f,g,i]	41.15 ± 0.96[a,b,c,e,f,g,i]
	400	25.92 ± 0.18[a,b,c,d,f,g,h,i]	47.96 ± 0.37[a,b,c,d,f,g,h,i]
	600	22.67 ± 0.18[a,b,c,d,e,g,h,i]	54.48 ± 0.37[a,b,c,d,e,g,h,i]
Experimental group (EMFB)	200	32.16 ± 0.28[a,b,c,d,e,f,h,i]	35.43 ± 0.56[a,b,c,d,e,f,h,i]
	400	28.80 ± 0.45[a,b,c,e,f,g,i]	42.17 ± 0.91[a,b,c,e,f,g,i]
	600	18.62 ± 0.14[a,b,c,d,e,f,g,h]	62.61 ± 0.27[a,b,c,d,e,f,g,h]

The data are represented as the mean ± SEM ($n = 5$ per group).

[a] compared to the negative control group receiving vehicle solvent (7% Tween 80 and 3% ethanol in distilled water); [b] compared to the positive control group receiving ARS at 6 mg/kg body weight; [c] compared to the positive control group receiving CQ at 25 mg/kg body weight; [d] compared to the experimental group receiving EMFL at 200 mg/kg body weight; [e] compared to the experimental group receiving EMFL at 400 mg/kg body weight; [f] compared to the experimental group receiving EMFL at 600 mg/kg body weight; [g] compared to the experimental group receiving EMFB at 200 mg/kg body weight; [h] compared to the experimental group receiving EMFB at 400 mg/kg body weight; [i] compared to the experimental group receiving EMFB at 600 mg/kg body weight. $p < 0.05$ was considered statistically significant.

The abbreviations are as follows: ARS represents artesunate; CQ represents chloroquine; EMFL represents the ethanolic extract of *M. ferrea* L. leaves; and EMFB represents the ethanolic extract of *M. ferrea* L. branches.

Table 1.2 Effects of the EMFL and EMFB on food and water consumption in the acute oral toxicity test. (Data presented corresponds to Figure 3)

Group	Dose (mg/kg)	Food consumption		Water consumption	
		Week 1 (g)	Week 2 (g)	Week 1 (mL)	Week 2 (mL)
Control group	-	28.50 ± 3.50	29.50 ± 0.50	53.00 ± 3.00	59.00 ± 6.00
Negative control group	-	27.00 ± 1.00	27.50 ± 0.50	54.50 ± 4.50	54.00 ± 2.00
Experimental group (EMFL)	2,000	27.50 ± 2.50	26.50 ± 1.50	52.00 ± 4.00	48.50 ± 4.50
Experimental group (EMFB)	2,000	26.00 ± 6.00	26.50 ± 3.50	62.50 ± 2.50	64.00 ± 6.00

The data are represented as the mean ± SEM (n = 5 per group).

The abbreviations are as follows: EMFL represents the ethanolic extract of *M. ferrea* L. leaves, and EMFB represents the ethanolic extract of *M. ferrea* L. branches.

Table 1.3 Effects of the EMFL and EMFB on body weight changes before treatment (day 0) and after completion of experiments (day 14) in the acute oral toxicity test. (Data presented corresponds to Figure 4)

Group	Dose (mg/kg)	Mean bodyweight	
		Day 0 (g)	Day 14 (g)
Control group	-	40.95 ± 0.53	43.26 ± 0.70
Negative control group	-	41.11 ± 0.46	43.47 ± 1.30
Experimental group (EMFL)	2,000	38.96 ± 0.51	42.09 ± 0.51
Experimental group (EMFB)	2,000	39.95 ± 0.62	42.82 ± 0.66

The data are represented as the mean ± SEM (n = 5 per group).

The abbreviations are as follows: EMFL represents the ethanolic extract of *M. ferrea* L. leaves, and EMFB represents the ethanolic extract of *M. ferrea* L. branches.

Table 1.4 Effects of the EMFL and EMFB on liver and kidney functions in the acute oral toxicity test. **(Data presented corresponds to Figure 5)**

Parameters/group	Control group	Negative control group	Experimental group (EMFL 2,000 mg/kg)	Experimental group (EMFB 2,000 mg/kg)
Liver function parameters				
AST (U/L)	120.60 ± 2.11	111.20 ± 4.79	115.80 ± 6.88	110.80 ± 6.09
ALT (U/L)	36.60 ± 2.29[c]	39.00 ± 4.25[c]	54.60 ± 1.86[a,b,d]	37.80 ± 2.96[c]
ALP (U/L)	85.60 ± 5.35	85.00 ± 7.33	103.80 ± 3.40	103.00 ± 3.61
Kidney function parameters				
BUN (mg/dL)	23.00 ± 0.84	22.80 ± 1.16	23.00 ± 0.71	23.60 ± 0.68
Creatinine (mg/dL)	0.50 ± 0.02	0.56 ± 0.08	0.42 ± 0.05	0.54 ± 0.04

The data are represented as the mean ± SEM (n = 5 per group).

[a] compared to the control group; [b] compared to the negative control group receiving vehicle solvent (7% Tween 80 and 3% ethanol in distilled water); [c] compared to the experimental group receiving EMFL at 2,000 mg/kg body weight; [d] compared to the experimental group receiving EMFB at 2,000 mg/kg body weight. $p < 0.05$ is considered statistically significant.

The abbreviations are as follows: AST represents aspartate aminotransferase; ALT represents alanine aminotransferase; ALP represents alkaline phosphatase; BUN represents blood urea nitrogen; EMFL represents the ethanolic extract of *M. ferrea* L. leaves; and EMFB represents the ethanolic extract of *M. ferrea* L. branches.
