# Supplementary material for: Exploring the potential antimalarial properties, safety profile, and phytochemical composition of Mesua ferrea Linn
Source: PLoS One. 2024 Dec 2;19(12):e0312047. doi: 10.1371/journal.pone.0312047 (PMC11611102; doi:10.1371/journal.pone.0312047)
Supplement: S2 File — (PDF) [file pone.0312047.s002.pdf]

Supporting information file 1.2

Histopathological examinations of the liver and kidney tissues in an acute oral toxicity test (uncropped raw images)

A. Liver tissues

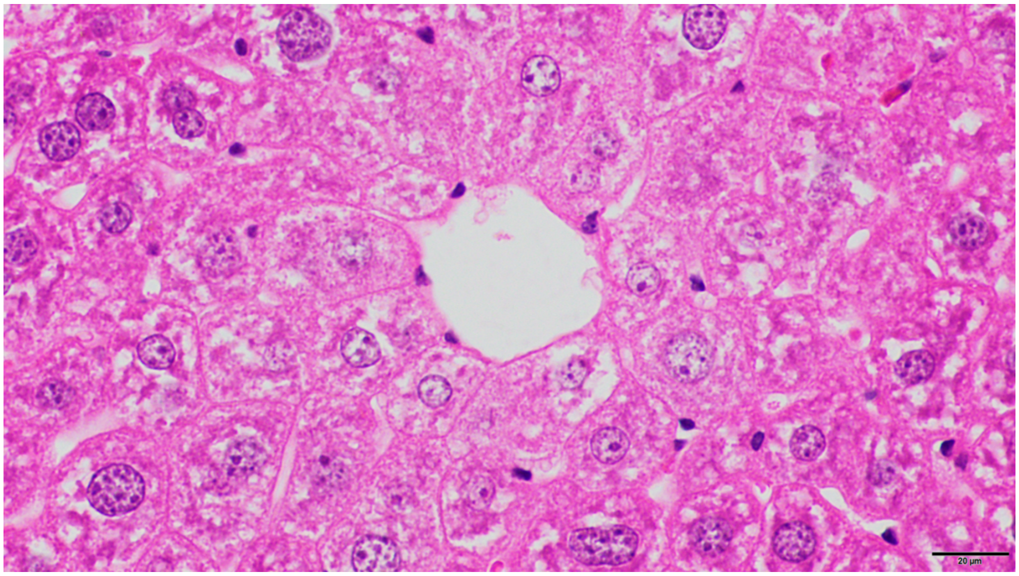

Control group

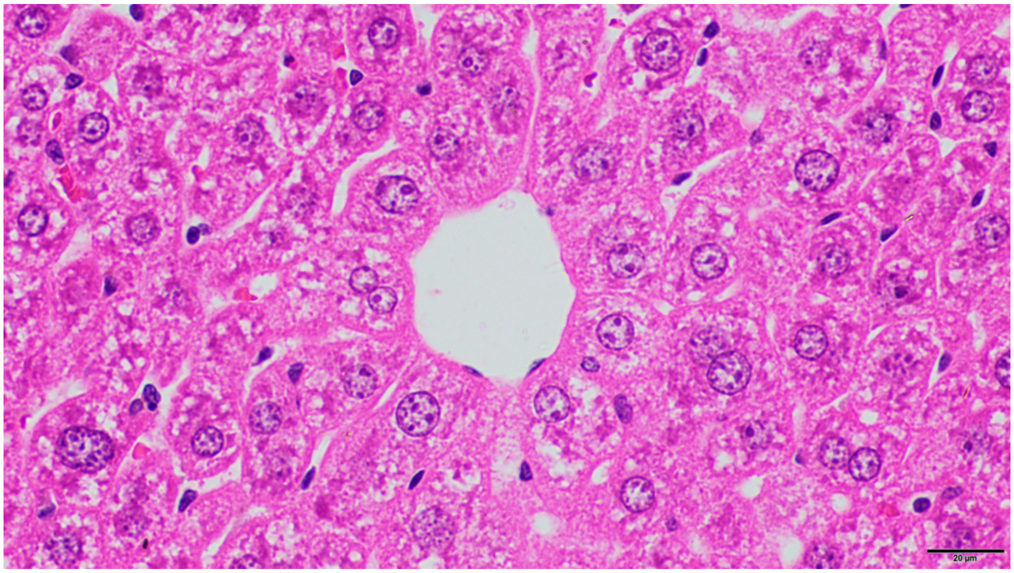

Negative control group (7% tween 80 and 3% ethanol in distilled water)

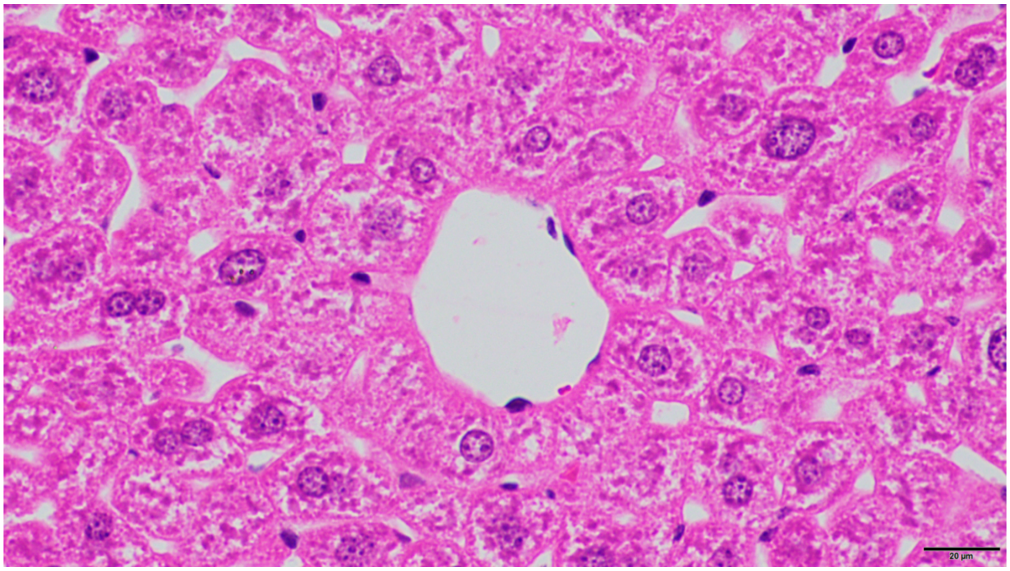

Experimental group (2,000 mg/kg body weight of EMFL)

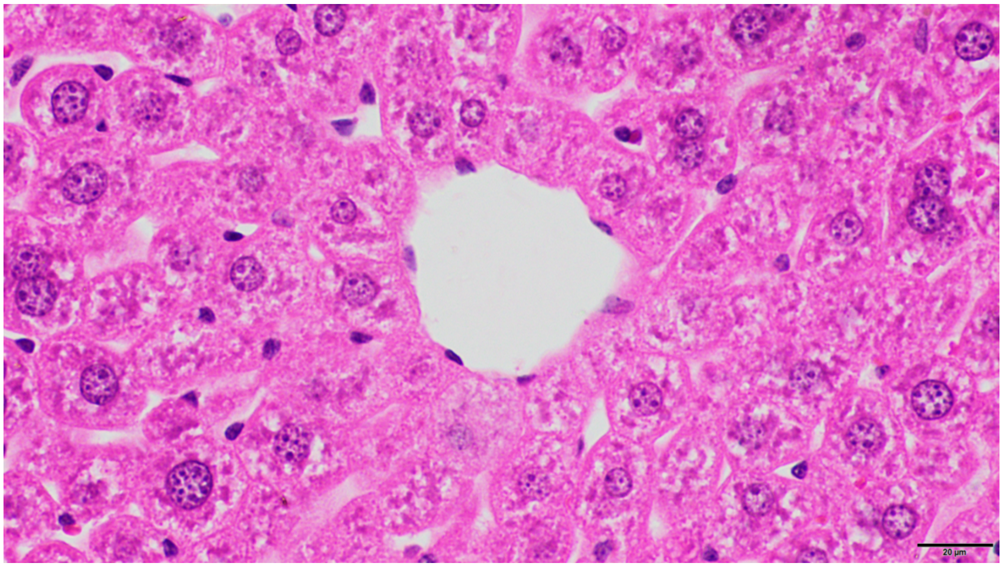

Experimental group (2,000 mg/kg body weight of EMFB)

*The abbreviations are as follows: EMFL represents the ethanolic extract of *M. ferrea* L. leaves, and EMFB represents the ethanolic extract of *M. ferrea* L. branches.

B. Kidney tissues

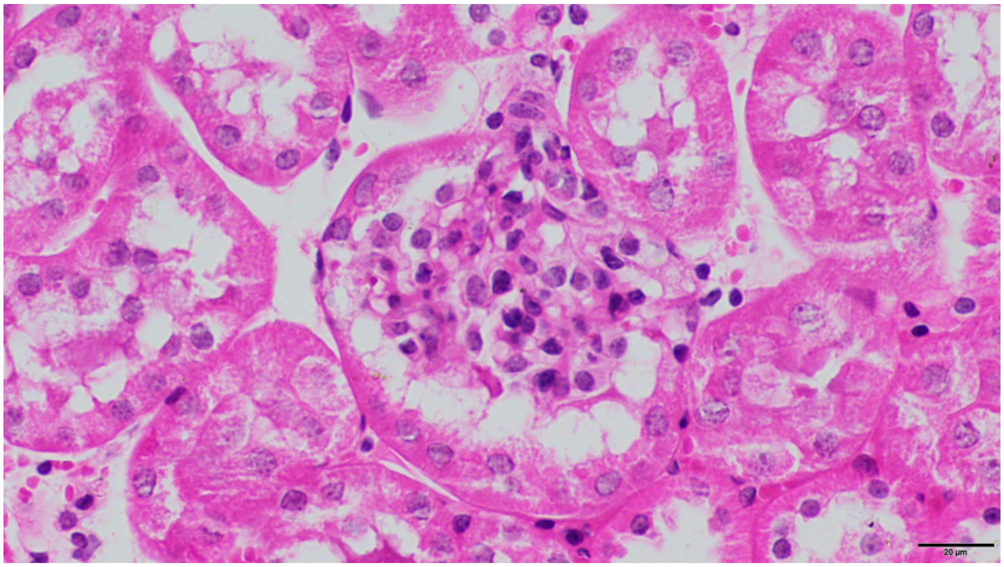

Control group

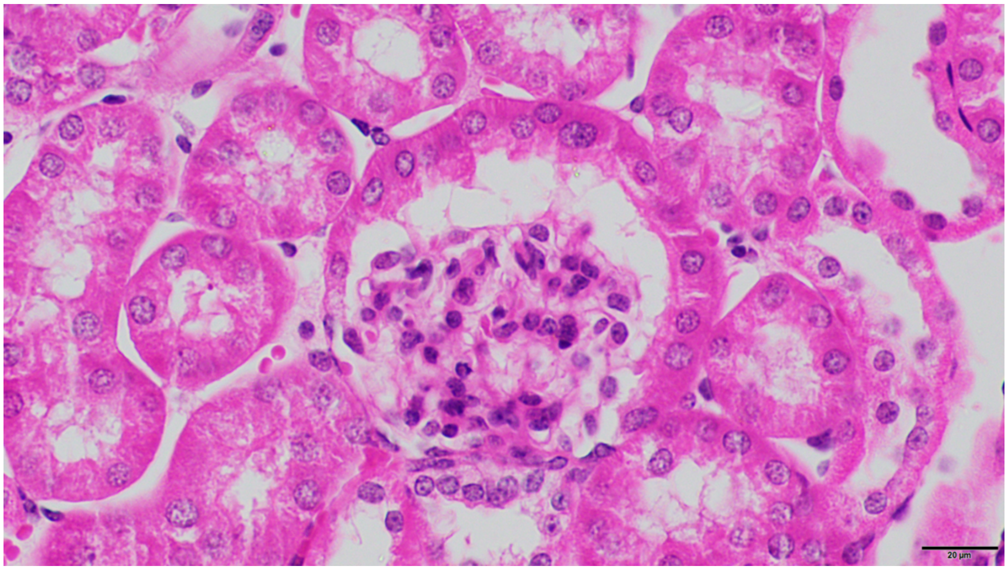

Negative control group (7% tween 80 and 3% ethanol in distilled water)

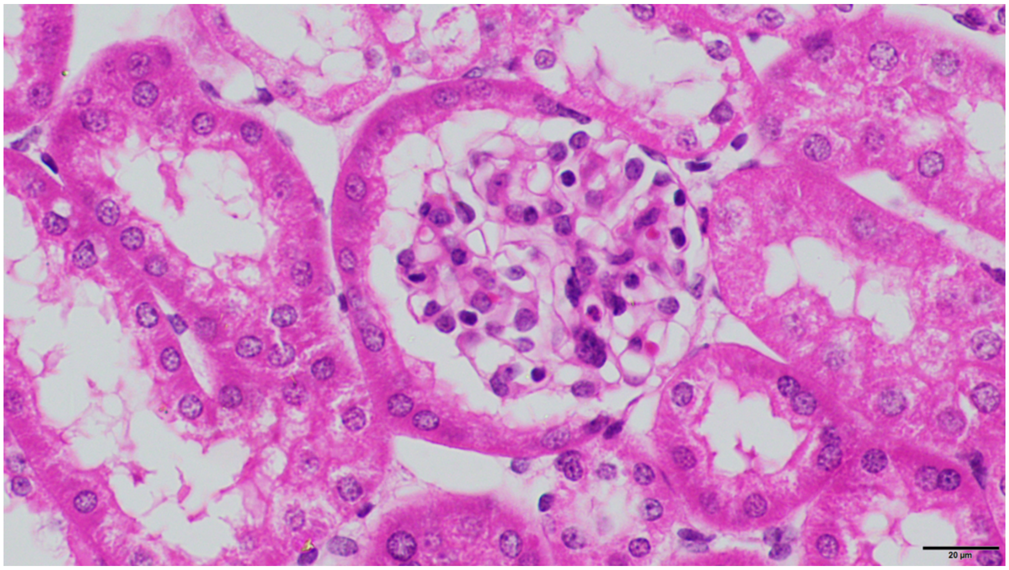

Experimental group (2,000 mg/kg body weight of EMFL)

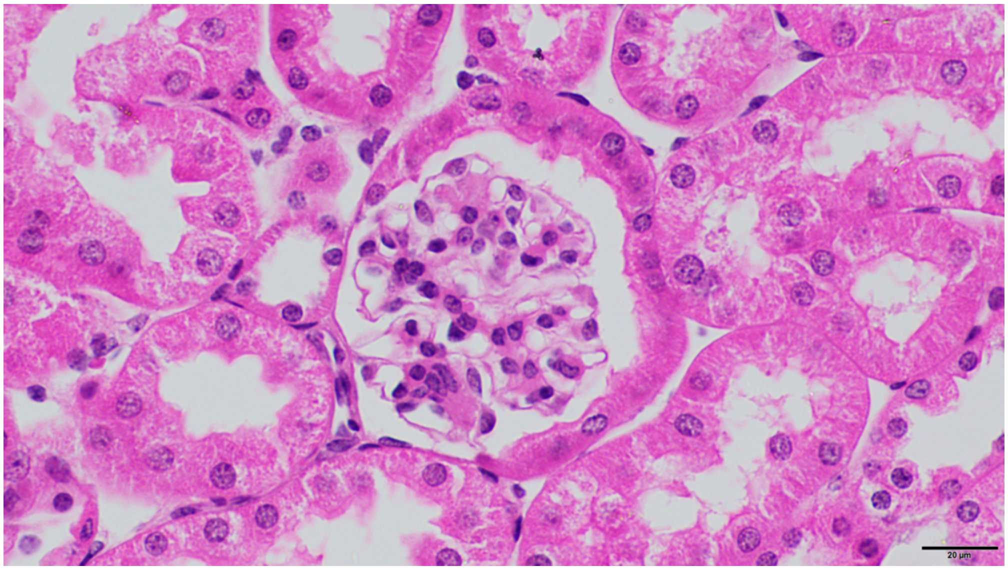

Experimental group (2,000 mg/kg body weight of EMFB)

*The abbreviations are as follows: EMFL represents the ethanolic extract of *M. ferrea* L. leaves, and EMFB represents the ethanolic extract of *M. ferrea* L. branches.
