# Supplementary material for: Exploring the potential antimalarial properties, safety profile, and phytochemical composition of Mesua ferrea Linn
Source: PLoS One. 2024 Dec 2;19(12):e0312047. doi: 10.1371/journal.pone.0312047 (PMC11611102; doi:10.1371/journal.pone.0312047)
Supplement: Attachment — Submitted filename: Point by point_12.08.2024.pdf [file pone.0312047.s004.pdf]

Point-by-point responses to the reviewers' comments

Additional Editor Comments:

This paper has unclear methodology and needs an overall elaboration to meet the publication quality.

Please also clarify the following:

1. How was the sample size of the animal experiments determined and which statistical test were used.

Response: In this study, we employed G*Power software version 3.1.9.6 [1] to calculate the required sample sizes for the *in vivo* antimalarial activity evaluated through a 4-day suppressive test. The statistical analysis conducted in G*Power software employed an "ANOVA: fixed effect, omnibus, one-way analysis" with the type of power analysis set to "A priori: compute required sample size - given α, power, and effect size" in accordance with previous studies [2]. The effect size was set at 0.8, the α error prob at 0.05, and the power ($1-\beta$ error prob) at 0.95, indicating a statistical power of 95%. With nine groups defined, this resulted in a minimum required sample size of forty-five experimental mice, allocated as five mice per group, as indicated in the manuscript; please see pages 14–15 and lines 309–319. For the acute oral toxicity test, we adhered to the Organization for Economic Cooperation and Development (OECD), test guideline no. 425 [3], which recommends using five mice per group for limit tests at a dosage of 2,000 mg/kg body weight. Consequently, we employed four groups of mice for this test, resulting in a total of twenty experimental mice, as indicated in the manuscript; please see pages 16 and lines 345–349. The sample size calculations and the use of animals in this study were approved by the Walailak University Institutional Animal Care and Use Committee (WU-IACUC) under the approval number WU-ACUC-66019.

References:

1. Faul F, Erdfelder E, Buchner A, Lang A-G. Statistical power analyses using G*Power 3.1: Tests for correlation and regression analyses. Behav Res Methods. 2009;41(4):1149-60. doi: 10.3758/BRM.41.4.1149.

2. Amadi PU, Agomuo EN, Ukaga CN, Njoku UC, Amadi JA, Nwaekpe CG. Preclinical trial of traditional plant remedies for the treatment of complications of gestational Malaria. Medicines (Basel). 2021;8(12). Epub 20211217. doi: 10.3390/medicines8120079. PubMed PMID: 34940291; PubMed Central PMCID: PMCPMC8703497.

3. OECD. Test No. 425: Acute Oral Toxicity: Up-and-Down Procedure2022.

2. State the name of the Institutional Animal Care and Use Committee (IACUC) or other relevant ethics board that reviewed the study protocol and formally accepted the study. As malaria infection model is a post procedure survival strategy the ethical clearance cannot be waived off.

Response: This study employed mouse models for both the 4-day suppressive test and the acute oral toxicity test. Prior to conducting the animal experiments, the study protocol was submitted to the Animal Ethics Committee at Walailak University. After a comprehensive review, the animal ethical clearance was obtained from the Walailak University Institutional Animal Care and Use Committee (WU-IACUC), with the approval number WU-ACUC-66019. All procedures were conducted in accordance with relevant guidelines and regulations for the use of animals, in compliance with the Animal Research: Reporting of In Vivo Experiments (ARRIVE) guidelines. Furthermore, the information relevant to animal use has been included in the *PLOS ONE* Humane Endpoints Checklist and the ARRIVE Guidelines 2.0: Author Checklist, which were submitted alongside the manuscript. For information, please see pages 7 and lines 152–159.

3. Standard guidelines of the Organization for Economic Cooperation and Development does not amount to ethical clearance.

Response: The acute oral toxicity test is essential for determining a safer dose range to manage the clinical signs and symptoms associated with drug administration [4]. To minimize the number of animals required for testing and to reduce animal stress, the OECD has established test guidelines, including test guideline no. 425 [3, 5], which was accepted as an alternative to the median lethal dose (LD_{50}) method in 2008 and revised in 2022 [6]. This guideline is widely recognized as a standard protocol for acute oral toxicity testing and is accepted by U.S. and international regulatory authorities, including the European Chemical Agency (ECHA), as an alternative method for chemical safety testing [7, 8]. In this study, we submitted study protocols relevant to the acute oral toxicity tests, adhering to OECD guidelines, to the Animal Ethics Committee at Walailak University. Following a thorough review, we obtained animal ethical clearance from the Walailak University Institutional Animal Care and Use Committee (WU-IACUC), with the approval number WU-ACUC-66019. Additionally, our literature review indicates that several studies have successfully utilized OECD guidelines for acute oral toxicity tests of various test substances [4, 9-11]. For information on animal ethics and the OECD guidelines, please see pages 7 (lines 152–159) and pages 16 (lines 343–345).

References:

3. OECD. Test No. 425: Acute Oral Toxicity: Up-and-Down Procedure2022.

4. Saleem U, Amin S, Ahmad B, Azeem H, Anwar F, Mary S. Acute oral toxicity evaluation of aqueous ethanolic extract of *Saccharum munja* Roxb. roots in albino mice as per OECD 425 TG. Toxicol Rep. 2017;4:580-5. Epub 20171031. doi: 10.1016/j.toxrep.2017.10.005. PubMed PMID: 29152463; PubMed Central PMCID: PMCPMC5671618.

5. Kojima H, Nakada T, Yagami A, Todo H, Nishimura J, Yagi M, et al. A step-by-step approach for assessing acute oral toxicity without animal testing for additives of quasi-

drugs and cosmetic ingredients. Curr Res Toxicol. 2023;4:100100. Epub 20221223. doi: 10.1016/j.crtox.2022.100100. PubMed PMID: 36619289; PubMed Central PMCID: PMCPMC9816657.

6. Sewell F, Ragan I, Horgan G, Andrew D, Holmes T, Manou I, et al. New supporting data to guide the use of evident toxicity in acute oral toxicity studies (OECD TG 420). Regul Toxicol Pharmacol. 2024;146:105517. Epub 20231012. doi: 10.1016/j.yrtph.2023.105517. PubMed PMID: 37838350.

7. Alternative methods accepted by US agencies. Available from: https://ntp.niehs.nih.gov/whatwestudy/niceatm/accept-methods.

8. Alternative methods for toxicity testing. Available from: https://joint-research-centre.ec.europa.eu/reference-measurement/european-union-reference-laboratories/eu-reference-laboratory-alternatives-animal-testing-eurl-ecvam/alternative-methods-toxicity-testing/validated-test-methods-health-effects/acute-toxicity_en.

9. Rocha JMV, de Souza VB, Panunto PC, Nicolosi JS, da Silva EDN, Cadore S, et al. *In vitro* and *in vivo* acute toxicity of a novel citrate-coated magnetite nanoparticle. PLoS One. 2022;17(11):e0277396. Epub 20221117. doi: 10.1371/journal.pone.0277396. PubMed PMID: 36395271; PubMed Central PMCID: PMCPMC9671459.

10. Uppu DS, Manjunath GB, Yarlagadda V, Kaviyil JE, Ravikumar R, Paramanandham K, et al. Membrane-active macromolecules resensitize NDM-1 gram-negative clinical isolates to tetracycline antibiotics. PLoS One. 2015;10(3):e0119422. Epub 20150319. doi: 10.1371/journal.pone.0119422. PubMed PMID: 25789871; PubMed Central PMCID: PMCPMC4366164.

11. Mulatu A, Megersa N, Tolcha T, Alemu T, Vetukuri RR. Antifungal compounds, GC-MS analysis and toxicity assessment of methanolic extracts of *Trichoderma* species in an animal model. PLoS One. 2022;17(9):e0274062. Epub 20220923. doi: 10.1371/journal.pone.0274062. PubMed PMID: 36149851; PubMed Central PMCID: PMCPMC9506656.

Reviewer #1: Exploring the potential antimalarial properties, safety profile, and phytochemical composition of *Mesua ferrea* Linn.

This study has highlighted the importance of phytochemicals to combat malarial parasite. The study investigated the therapeutic potential of *Mesua ferrea* Linn., against *Plasmodium falciparum*'s resistance to lead drug resistance to global malaria control. *In vitro* assays evaluated with crude extracts from *M. ferrea* leaves and branches shows impressive data (4.54 µg/mL), followed by the ethanolic extract of leaves. Besides the study has been replicated in mouse model holds its promising antimalarial activity. Overall, this study highlights the potential of *M. ferrea* as a natural antimalarial resource.

However, these are some aspects which authors should address:

1. In the material and methods section the human ethical clearance is stated. However, no such data has been presented in this study. It is unnecessary and should be removed.

Response: In this study, we utilized human blood samples from participants. Erythrocytes were isolated from blood samples and subsequently used for the *in vitro* cultivation of the *Plasmodium falciparum* K1 strain to evaluate the antimalarial activity through the parasite lactate dehydrogenase (pLDH) assay, as detailed on pages 9 and lines 188–190. Furthermore, we have included a statement regarding the ethical clearance for the use of human blood samples in the ethics approval section; please see pages 7 and lines 144–150.

2. All the data have been presented in form of table. These can be made into graphical representation. This would increase the readability of the article.

Response: Thank you very much for your valuable comments. In response to your recommendations, we have made revisions to improve the clarity and readability of our manuscript. Specifically, we have transformed tables 5, 6, 7, and 8 into figures 2, 3, 4, and 5, respectively, to enhance the visual representation of our findings and facilitate easier comprehension. Please see pages 26–27 (lines 504–516), pages 28 (lines 538–553), pages 29 (lines 560–570), and pages 30–31 (lines 585–595) respectively. However, upon further evaluation, we concluded that tables 1, 2, 3, and 4 were not suitable for conversion into graphical format. Therefore, we have retained these tables in their original format to maintain optimal readability and ensure that the information is easily understood by the reader.

3. The arrows in the histological section should be made bold.

Response: Thank you very much for your valuable comments. I have improved figure 6 by enlarging and bolding the arrows, letter symbols, and descriptions related to the histopathological examination of the liver and kidney tissues in order to enhance readability. Please see figure 6, pages 32–33 and lines 615–622.

4. Further, *Plasmodium berghei* ANKA is a model for cerebral malaria. Authors may discuss the effect of extracts on the brain inflammation.

Response: Thank you very much for your valuable comments. In response to your recommendations, I have included a discussion of several compounds reported to exhibit protective effects on the brain during *P. berghei* ANKA infections, specifically those identified in the ethanolic extracts of *M. ferrea* L. leaves and branches (EMFL and EMFB). For instance, the diterpenoid phytol present in EMFL has been shown to have protective effects against brain damage induced by *P. berghei* infection [12]. Additionally, eugenol, a derivative of methoxyeugenol present in EMFB, has been reported to prevent cerebral edema and cognitive dysfunction associated with the *P. berghei* ANKA infection [13]. Please see discussion section on pages 40 (lines 792–795) and pages 41 (lines 810–812). Furthermore, several compounds and subclasses identified in EMFL and EMFB demonstrate anti-inflammatory activity, as discussed in the relevant section. These include friedelin, betulin, methoxyeugenol, linoleic acid, anthraquinone, and terpenoids. The presence of these compounds in the extracts may contribute

to the mitigation of the inflammatory response during *P. berghei* ANKA infections, either individually or through synergistic interactions.

References:

12. Usman MA, Usman FI, Abubakar MS, Salman AA, Adamu A, Ibrahim MA. Phytol suppresses parasitemia and ameliorates anaemia and oxidative brain damage in mice infected with *Plasmodium berghei*. Exp Parasitol. 2021;224:108097. Epub 2021/03/20. doi: 10.1016/j.exppara.2021.108097. PubMed PMID: 33736972.

13. Pontes KAO, Silva LS, Santos EC, Pinheiro AS, Teixeira DE, Peruchetti DB, et al. Eugenol disrupts *Plasmodium falciparum* intracellular development during the erythrocytic cycle and protects against cerebral malaria. Biochim Biophys Acta Gen Subj. 2021;1865(3):129813. Epub 20201213. doi: 10.1016/j.bbagen.2020.129813. PubMed PMID: 33321150.

5. The peak area represents the abundance of the compound in the extract. Compounds may be listed in the order of their abundance

Response: Thank you very much for your valuable comments. In response, I have rearranged the order of the compounds identified in the ethanolic extracts of *M. ferrea* L. leaves and branches (EMFL and EMFB) through gas chromatography-mass spectrometry (GC-MS) analysis. The compounds are now presented in descending order based on their abundance, as determined by peak area. Please see tables 3 (pages 23–24, lines 481–483) and 4 (pages 24–25, lines 485–487).

6. The study shows promising results and is well written with relevant references.

Response: Thank you very much.

Reviewer #2: Atthaphon Konyanee et al 'Exploring the potential antimalarial properties, safety profile, and phytochemical composition of *Mesua ferrea* Linn. Here are some constructive comments and observations you might consider for the provided results:

1. Line 148-149 It's important to explicitly state whether written or verbal informed consent was obtained from participants. Please clarify this detail.

Response: In this study, we utilized human blood samples from participants for the *in vitro* cultivation of the human malaria *P. falciparum* K1 strain. Prior to blood sample collection, our study protocol was approved by the Ethics Committee in Human Research Walailak University (approval number: WUEC-23-062-01). We also provided participants with an information sheet outlining the details of the study, along with an informed consent form. After reviewing this information, participants who agreed to take part in the study provided written informed consent.

Blood samples were then collected by an expert medical technologist, as described in the ethics approval and consent participation section. Please see pages 7 and lines 144–150.

2. Could you provide more information on the anesthesia protocols and criteria for humane endpoints?

Response: In this study, we employed the inhalation method to induce anesthesia in mice using 2% isoflurane (Piramal Critical Care, PA, USA) mixed with oxygen as the carrier gas within an anesthesia induction chamber, following an established protocol [14]. This approach aimed to minimize potential distress in the mice. To verify successful anesthesia induction, we assessed the mice by evaluating their toe-pinch pain reflex, following an established protocol [15]. Additionally, the animals were routinely monitored twice daily, and any animals displaying signs of coma or experiencing severe symptoms, including immobility, lack of body extension, and unresponsiveness to external stimuli, were immediately euthanized in a humane manner to alleviate pain and distress [16]. Euthanasia was performed under deep isoflurane anesthesia, in accordance with the established protocol [16]. Please see pages 13–14 (lines 292–297), pages 14 (lines 301–305), pages 15 (lines 329–331), and pages 16 (lines 358–359), respectively. Moreover, we have also included this information in the *PLOS ONE* Humane Endpoints Checklist document.

References:

14. Ebert G, Lopaticki S, O'Neill MT, Steel RWJ, Doerflinger M, Rajasekaran P, et al. Targeting the extrinsic pathway of hepatocyte apoptosis promotes clearance of *Plasmodium* liver infection. Cell Rep. 2020;30(13):4343-54.e4. doi: 10.1016/j.celrep.2020.03.032. PubMed PMID: 32234472.

15. Sarfo BY, Armah HB, Irune I, Adjei AA, Olver CS, Singh S, et al. *Plasmodium yoelii* 17XL infection up-regulates RANTES, CCR1, CCR3 and CCR5 expression, and induces ultrastructural changes in the cerebellum. Malar J. 2005;4:63. Epub 20051216. doi: 10.1186/1475-2875-4-63. PubMed PMID: 16359553; PubMed Central PMCID: PMCPMC1343570.

16. Matsubara R, Aonuma H, Kojima M, Tahara M, Andrabi SB, Sakakibara H, et al. Plant hormone salicylic acid produced by a malaria parasite controls host immunity and cerebral malaria outcome. PLoS One. 2015;10(10):e0140559. Epub 20151014. doi: 10.1371/journal.pone.0140559. PubMed PMID: 26466097; PubMed Central PMCID: PMCPMC4605785.

3. Could you specify if any permits or ethical considerations were required for the collection of *M. ferrea* L. leaves and branches?

Response: The plant samples utilized in this study comprise the mature leaves and branches of *Mesua ferrea* Linn. The *M. ferrea* L. tree was located at Walailak University in Nakhon Si Thammarat, Thailand, and samples were collected in March 2022 (GPS coordinates: 8°38'34.8"N, 99°53'58.9"E). Prior to sample collection, official documentation was submitted to the Division of Landscape Architecture and Environment (DLE) at Walailak University to obtain the necessary

permissions for the collection of 500 grams of *M. ferrea* L. leaves and branches, in accordance with university regulations. Following the approval from the DLE, we collected the samples, which included 500 grams of *M. ferrea* L. leaves and branches for use in the extraction process.

4. Can you provide more details on the statistical methods used to analyze the data, particularly in terms of the ANOVA model and post hoc tests applied? Additionally, were power analyses conducted to determine the sample sizes?

Response: In this study, we employed one-way analysis of variance (ANOVA) to assess overall differences in means among multiple groups. A post hoc Turkey's multiple comparison test was subsequently utilized to determine which specific pairs of means differed following a significant ANOVA result. This analysis was applied to various measured parameters, including percentage parasitemia, percentage parasitemia suppression, food consumption, water consumption, mean body weight, and liver and kidney function parameters. A *p*-value of less than 0.05 ($p < 0.05$) was considered statistically significant. Additionally, this statistical testing methodology has been employed in several previous studies relevant to the testing of antimalarial activity [17-20]. Please see pages 17−18 and lines 383−390. In addition, we utilized G*Power software version 3.1.9.6 [1] to calculate the required sample size for the *in vivo* antimalarial activity assessed through a 4-day suppressive test. The statistical analysis conducted in G*Power employed the "ANOVA: fixed effect, omnibus, one-way analysis" and set power (1−β error prob) at 0.95, which indicates a statistical power of 95% for the sample size calculations in this study. Further details regarding this calculation are provided in response to question 1 in the editor's comments.

References:

1. Faul F, Erdfelder E, Buchner A, Lang A-G. Statistical power analyses using G*Power 3.1: Tests for correlation and regression analyses. Behav Res Methods. 2009;41(4):1149-60. doi: 10.3758/BRM.41.4.1149.

17. Muluye AB, Desta AG, Abate SK, Dano GT. Anti-malarial activity of the root extract of *Euphorbia abyssinica* (*Euphorbiaceae*) against *Plasmodium berghei* infection in mice. Malar J. 2019;18(1):261. Epub 20190730. doi: 10.1186/s12936-019-2887-7. PubMed PMID: 31362744; PubMed Central PMCID: PMCPMC6668069.

18. Misganaw D, Amare GG, Mengistu G. Chemo suppressive and curative potential of *Hypoestes forskalei* against *Plasmodium berghei*: Evidence for *in vivo* antimalarial activity. J Exp Pharmacol. 2020;12:313-23. Epub 20200907. doi: 10.2147/jep.S262026. PubMed PMID: 32982487; PubMed Central PMCID: PMCPMC7498819.

19. Ayalew M, Atnafie SA, Bekele A. Antimalarial activity of solvent fractions of a leaf of *Eucalyptus globulus labill* against *Plasmodium berghei* infected mice. BMC Complement Med Ther. 2022;22(1):221. Epub 20220816. doi: 10.1186/s12906-022-03702-1. PubMed PMID: 35974350; PubMed Central PMCID: PMCPMC9380366.

20. Birru EM, Geta M, Gurmu AE. Antiplasmodial activity of *Indigofera spicata* root extract against *Plasmodium berghei* infection in mice. Malar J. 2017;16(1):198. Epub

20170516. doi: 10.1186/s12936-017-1853-5. PubMed PMID: 28511659; PubMed Central PMCID: PMCPMC5434560.

5. It would strengthen the manuscript to include more explicit details on the protocols used, including any modifications from previously established methods. This would enhance transparency and facilitate replication of the study.

Response: Thank you very much for your valuable comments. In this study, we employed several protocols, some of which were modified, and we have added more detailed descriptions of these methods in the manuscript (Methods section) to enhance transparency and facilitate the replication of our study, as recommended by the reviewer.

- **Plant material and extraction procedure:** We have provided additional details regarding the selection criteria for the plant specimens, specifically the leaves and branches of *M. ferrea* L. Please see pages 8 and lines 162–164.
- **Evaluation of *in vitro* antimalarial activity using the parasite lactate dehydrogenase (pLDH) assay:** We have included more specific information about the volumes of aliquots taken from lysed erythrocytes, as well as the quantities of Malstat reagent and the *p*-nitroblue tetrazolium chloride and phenazine ethosulfate (NBT/PES) solution used to measure the pLDH reaction. Please see pages 10 and lines 216–220.
- **Evaluation of *in vitro* cytotoxicity using the 3-(4,5-dimethylthiazol-2-yl)-2,5-diphenyltetrazolium bromide (MTT) assay:** We have added detailed information regarding the volumes of MTT solution and dimethyl sulfoxide (DMSO) used in the assay, as well as the two specific absorbance values measured during the reaction. Please see pages 11 and lines 238–246.
- ***In vivo* acute oral toxicity:** We have included more detailed information regarding OECD guidelines no. 425, which were employed in this study. Please see pages 16 and lines 343–345.
- **Remaining methods:** We have ensured that all other methods used in this study are described in detail.

6. In the acute oral toxicity test, how were the criteria for assessing toxicity determined, and what were the specific endpoints used for determining humane euthanasia?

Response: In this study, we investigated acute oral toxicity in mice following treatment with plant extracts by observing various gross physical and behavioral changes, including rigidity, sleep, diarrhea, depression, abnormal secretion, and hair erection, within 3 hours post-treatment. The mice were monitored twice daily for a duration of 14 days. Additionally, we assessed food and water consumption behaviors throughout this period and measured body weight on Day 0 (prior to treatment) and Day 14 (at the conclusion of the study). During the experiments, any animals exhibiting signs of coma or severe symptoms, including immobility, lack of body extension, and unresponsiveness to external stimuli, were immediately euthanized in a humane manner using deep isoflurane anesthesia to alleviate pain and distress [16]. Please see pages 16 (lines 349–358) and pages 14 (lines 301–305), respectively. Moreover, we have also included this information in the *PLOS ONE* Humane Endpoints Checklist document.

Reference:

16. Matsubara R, Aonuma H, Kojima M, Tahara M, Andrabi SB, Sakakibara H, et al. Plant hormone salicylic acid produced by a malaria parasite controls host immunity and cerebral malaria outcome. PLoS One. 2015;10(10):e0140559. Epub 20151014. doi: 10.1371/journal.pone.0140559. PubMed PMID: 26466097; PubMed Central PMCID: PMCPMC4605785.

7. Given the detailed methodology for plant extraction, it would be beneficial to include any information regarding potential conflicts of interest in the selection and processing of *M. ferrea* L. leaves and branches.

Response: Thank you very much for your valuable comments. In our selection of *M. ferrea* L. leaves and branches for extraction, we specifically selected only mature leaves, characterized by their blue-grey to dark green coloration, for extraction [21]. For the branches, we selected mature branches, which are defined as those parts that grow out from the trunk and are characterized by a reddish-brown. The extraction process was conducted in accordance with established protocols [22, 23], and we have provided all detailed information regarding the extraction methods used for *M. ferrea* L. leaves and branches, including both maceration and decoction methods, in the manuscript. To ensure transparency and avoid any potential conflicts of interest regarding the selection and extraction processes, we have clearly outlined these procedures in the manuscript. Please see pages 8 and lines 162−184.

References:

21. Asif M, Jafari SF, Iqbal Z, Revadigar V, Oon CE, Majid ASA, et al. Ethnobotanical and phytopharmacological attributes of *Mesua ferrea*: A mini review. J Appl Pharm Sci. 2017;7:242-51.

22. Kaewdana K, Chaniad P, Jariyapong P, Phuwajaroanpong A, Punsawad C. Antioxidant and antimalarial properties of *Sophora exigua* Craib. root extract in *Plasmodium berghei*-infected mice. Trop Med Health. 2021;49(1):24. Epub 20210319. doi: 10.1186/s41182-021-00314-2. PubMed PMID: 33741053; PubMed Central PMCID: PMCPMC7980637.

23. Chaniad P, Techarang T, Phuwajaroanpong A, Plirat W, Viriyavejakul P, Septama AW, et al. Antimalarial efficacy and toxicological assessment of medicinal plant ingredients of Prabchompoothaweep remedy as a candidate for antimalarial drug development. BMC Complement Med Ther. 2023;23(1):12. Epub 20230118. doi: 10.1186/s12906-023-03835-x. PubMed PMID: 36653791; PubMed Central PMCID: PMCPMC9847039.

8. Are there studies or hypotheses regarding the mechanism by which the ethanolic extracts exert their antimalarial activity? How do these mechanisms relate to the identified phytochemicals?

Response: Thank you very much for your valuable comments. In our previous study, we demonstrated that the ethanolic extract of *M. ferrea* L. flowers, a component of the Pra-Sa-Chan-

Dang (PSCD) remedy used in Thai traditional medicine, exhibited antimalarial activity against the *P. falciparum* K1 strain, with an IC_{50} value of 4.30 ± 0.73 μg/mL [24]. Please see the introduction section on pages 6 and lines 124−127. Furthermore, other studies have provided detailed gas chromatography-mass spectrometry (GC-MS) profiles of the ethanolic extract of *M. ferrea* L. flower buds, identifying the main phytochemical compounds. The main compounds identified include eugenol (61.49%), cinnamaldehyde (15.15%), terpinyl acetate (7.97%), 2-methoxycinnamaldehyde (2.57%), palmitic acid (2.37%), 2H-1-benzopyran (1.91%), and caryophyllene (1.06%), based on their respective peak areas [25]. Eugenol has been reported to exhibit antimalarial activity against the *P. falciparum* W2 strain, with an IC_{50} value of 532.42 ± 29.55 μM. Its potential mechanism of action involves modifications to plasma membranes, including the nuclear and digestive vacuole membranes of parasite cells [13]. These findings suggest that compounds present in the ethanolic extract of *M. ferrea* L. flowers, such as eugenol, may contribute to the extract's antimalarial activity. Furthermore, previous studies have reported that the ethanolic extracts of *M. ferrea* L. leaves and stem bark exhibit antioxidant activity, which is associated with the presence of phenolic, alkaloid, and flavonoid compounds in the extracts [26, 27]. It is plausible that the compounds present in the ethanolic extracts of *M. ferrea* L. leaves and stem bark may contribute to their antimalarial activity through their antioxidant properties. Please see the discussion section on pages 34 and lines 644−653. According to our current study, we also identified several compounds in the ethanolic extract of *M. ferrea* L. leaves and branches (EMFL and EMFB) through GC-MS analysis. For instance, betulin, a triterpenoid identified in the EMFL, has been reported to exhibit antimalarial activity against the *P. falciparum* 3D7 strain, with an IC_{50} value of less than 27 μM, and its possible mechanism of action involves forming hydrogen bonds with the erythrocyte membrane, leading to structural changes that prohibit merozoite invasion and growth [28]. Additionally, oleamide, identified in both EMFL and EMFB, has been reported to display a relative binding affinity of 3.5-fold with *Plasmodium falciparum* thioredoxin reductase, which is consistent with its *in vitro* antimalarial activity against *P. falciparum* K1 with an IC_{50} value of 4.29 μg/mL [29]. Moreover, we have discussed other compounds identified in EMFL and EMFB, along with relevant references supporting their possible mechanisms of action and antimalarial activity. Please see pages 39–44 and lines 768–882.

References:

13. Pontes KAO, Silva LS, Santos EC, Pinheiro AS, Teixeira DE, Peruchetti DB, et al. Eugenol disrupts *Plasmodium falciparum* intracellular development during the erythrocytic cycle and protects against cerebral malaria. Biochim Biophys Acta Gen Subj. 2021;1865(3):129813. Epub 20201213. doi: 10.1016/j.bbagen.2020.129813. PubMed PMID: 33321150.

24. Chaniad P, Phuwajaroanpong A, Plirat W, Konyanee A, Septama AW, Punsawad C. Assessment of antimalarial activity of crude extract of Chan-Ta-Lee-La and Pra-Sa-Chan-Dang formulations and their plant ingredients for new drug candidates of malaria treatment: *In vitro* and *in vivo* experiments. PLoS One. 2024;19(1):e0296756. Epub 20240111. doi: 10.1371/journal.pone.0296756. PubMed PMID: 38206944; PubMed Central PMCID: PMCPMC10783769.

25. Perumal R, Vellingiri V, Natesan R, Pemaiah B. Investigation on pharmacognostic parameters of *Sirunagapoo* (*Mesua ferrea* L): A traditional Indian herbal drug. Pharmacogn J. 2019;11(2).

26. Prasad D N, Rao BG, Rao ES, Rao TM, Praneeth D VS. Quantification of phytochemical constituents and *in-vitro* antioxidant activity of *Mesua ferrea* leaves. Asian Pac J Trop Biomed. 2012;2(2, Supplement):S539-S42. doi: https://doi.org/10.1016/S2221-1691(12)60269-X.

27. Rajesh KP, Manjunatha H, Krishna V, Kumara Swamy BE. Potential *in vitro* antioxidant and protective effects of *Mesua ferrea* Linn. bark extracts on induced oxidative damage. Ind Crops Prod. 2013;47:186-98. doi: https://doi.org/10.1016/j.indcrop.2013.03.008.

28. Ziegler HL, Franzyk H, Sairafianpour M, Tabatabai M, Tehrani MD, Bagherzadeh K, et al. Erythrocyte membrane modifying agents and the inhibition of *Plasmodium falciparum* growth: structure–activity relationships for betulinic acid analogues. Bioorg Med Chem. 2004;12(1):119-27. doi: 10.1016/j.bmc.2003.10.010. PubMed PMID: 14697777.

29. Munigunti R, Nelson N, Mulabagal V, Gupta MP, Brun R, Calderón AI. Identification of oleamide in *Guatteria recurvisepala* by LC/MS-based *Plasmodium falciparum* thioredoxin reductase ligand binding method. Planta Med. 2011;77(15):1749-53. Epub 20110512. doi: 10.1055/s-0030-1271080. PubMed PMID: 21567357.

9. Please provide a clear Figure 2 with an arrow indicating the designated paraphrase area.

Response: Thank you very much for your valuable comments. I have enlarged and bolded the arrows, letter symbols, and descriptions in figure 2 (now revised to figure 6) to enhance clarity in the histopathological examinations of liver and kidney tissues, thereby improving overall readability. Please see figure 6 (pages 32, lines 615–622).

****
