# Supplementary material for: Exploring the potential antimalarial properties, safety profile, and phytochemical composition of Mesua ferrea Linn
Source: PLoS One. 2024 Dec 2;19(12):e0312047. doi: 10.1371/journal.pone.0312047 (PMC11611102; doi:10.1371/journal.pone.0312047)
Supplement: Attachment — Submitted filename: Exploring the potential antimalarial properties.docx [file pone.0312047.s003.docx]

Exploring the potential antimalarial properties, safety profile, and phytochemical composition of Mesua ferrea Linn.

This study has highlighted the importance of phytochemicals to combat malarial parasite. The study investigated the therapeutic potential of Mesua ferrea Linn., against Plasmodium falciparum’s resistance to lead drug resistance to global malaria control. In vitro assays evaluated with crude extracts from M. ferrea leaves and branches shows impressive data (4.54 µg/mL), followed by the ethanolic extract of leaves. Besides the study has been replicated in mouse model holds its promising antimalarial activity. Overall, this study highlights the potential of M. ferrea as a natural antimalarial resource

However, these are some aspects which authors should address:

1) In the material and methods section the human ethical clearance is stated. However, no such data has been presented in this study. It is unnecessary and should be removed.
2) All the data have been presented in form of table. These can be made into graphical representation. This would increase the readability of the article.
3) The arrows in the histological section should be made bold.
4) Further, Plasmodium berghei anka is a model for celebral malaria. Authors may discuss the effect of extracts on the brain inflammation.
5) The peak area represents the abundance of the compound in the extract. Compounds may be listed in the order of their abundance

The study shows promising results and is well written with relevant references.
